# Hypothermic manipulation of bone cement can extend the handling time during vertebroplasty

**DOI:** 10.1186/1471-2474-13-198

**Published:** 2012-10-16

**Authors:** Po-Liang Lai, Ching-Lung Tai, I-Ming Chu, Tsai-Sheng Fu, Lih-Huei Chen, Wen-Jer Chen

**Affiliations:** 1 Department of Orthopedic Surgery, Chang Gung Memorial Hospital, Chang Gung University College of Medicine, Taoyuan, Taiwan; 2 Graduate Institute of Medical Mechatronics, Department of Mechanical Engineering, Chang Gung University, Taoyuan, Taiwan; 3 Department of Chemical Engineering, National Tsing Hua University, Hsinchu, Taiwan

**Keywords:** Bone cement, Precooling, Ice bath, Working time, Handling time

## Abstract

**Background:**

Polymethylmethacrylate (PMMA) is commonly used for clinical applications. However, the short handling time increases the probability of a surgeon missing the crucial period in which the cement maintains its ideal viscosity for a successful injection. The aim of this article was to illustrate the effects a reduction in temperature would have on the cement handling time during percutaneous vertebroplasty.

**Methods:**

The injectability of bone cement was assessed using a cement compressor. By twisting the compressor, the piston transmits its axial load to the plunger, which then pumps the bone cement out. The experiments were categorized based on the different types of hypothermic manipulation that were used. In group I (room temperature, sham group), the syringes were kept at 22°C after mixing the bone cement. In group 2 (precooling the bone cement and the container), the PMMA powder and liquid, as well as the beaker, spatula, and syringe, were stored in the refrigerator (4°C) overnight before mixing. In group 3 (ice bath cooling), the syringes were immediately submerged in ice water after mixing the bone cement at room temperature.

**Results:**

The average liquid time, paste time, and handling time were 5.1 ± 0.7, 3.4 ± 0.3, and 8.5 ± 0.8 min, respectively, for group 1; 9.4 ± 1.1, 5.8 ± 0.5, and 15.2 ± 1.2 min, respectively, for group 2; and 83.8 ± 5.2, 28.8 ± 6.9, and 112.5 ± 11.3 min, respectively, for group 3. The liquid and paste times could be increased through different cooling methods. In addition, the liquid time (i.e. waiting time) for ice bath cooling was longer than for that of the precooling method (*p* < 0.05).

**Conclusions:**

Both precooling (i.e. lowering the initial temperature) and ice bath cooling (i.e. lowering the surrounding temperature) can effectively slow polymerization. Precooling is easy for clinical applications, while ice bath cooling might be more suitable for multiple-level vertebroplasty. Clinicians can take advantage of the improved injectability without any increased cost.

## Background

Osteoporotic vertebral fractures represent a major health care problem because they cause severe, debilitating back pain that consequently reduces physical function and enormously affects quality of life. Conservative management, including analgesics, bed rest, braces, and rehabilitation, are indicated for patients who do not have any neurological impairment [1]. Vertebroplasty has been widely accepted as a therapeutic strategy for painful osteoporotic compression fractures [2-5]. In this procedure, bone cement is percutaneously injected under pressure into a vertebra through a cannula. Polymerization of the bone cement stabilizes the fractured vertebra by increasing its mechanical strength, thereby providing symptomatic pain relief [2,6,7].

Polymethylmethacrylate (PMMA) is a type of bone cement that is frequently used in clinical applications. The bone cement begins curing at a rapid rate immediately after the liquid monomers and the powder polymers are mixed [8]. A crucial time frame exists in which the cement has the ideal viscosity for a successful injection, and a surgeon can miss this short handling window [9]. Several studies have reported complications caused by cement leakage, with subsequent neurological sequels [2,10,11].

It has been recognized that temperature reduction has a significant influence on cement polymerization time [12-15]. The average polymerization time is approximately 2-5 min, depending on the temperature and the specific brand of products used. The operator has limited time available to deliver the bone cement through the spinal cannula into the body. The aim of this article was to illustrate the effects a temperature reduction has on the cement handling time during a percutaneous vertebroplasty.

## Methods

**Figure 1:**
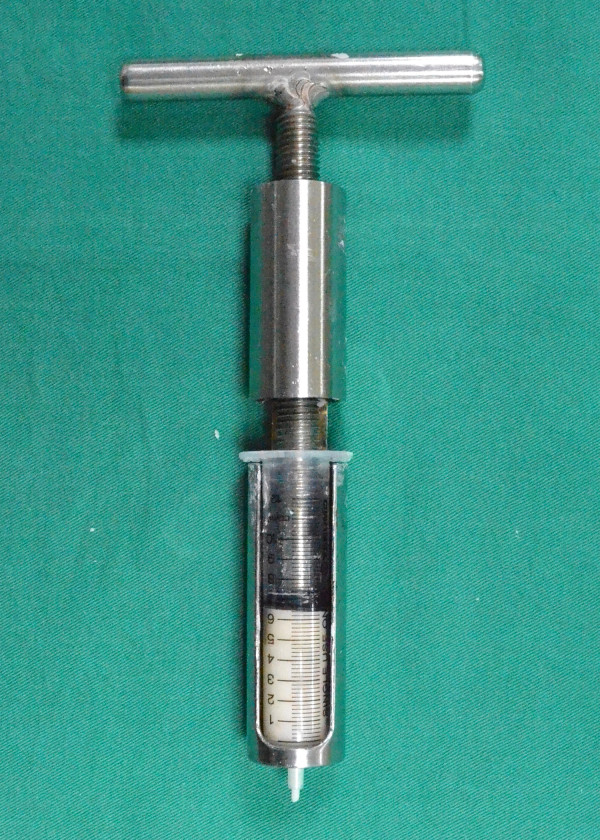
**A photograph of the bone cement compressor.** By twisting the compressor, the piston delivers its axial load to the plunger, which then pumps the bone cement out.

**Figure 2:**
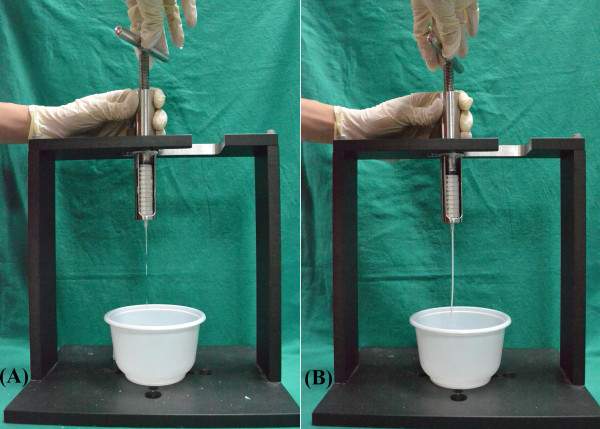
A photographic demonstration of the bone cement dripping from the syringe orifice during (A) the liquid phase and of the syringe pumping cement out without breaking its continuity during (B) the paste phase.

In this experiment, commercially available acrylic bone cement Simplex P (Stryker, US) was used. The technique for cement preparation consisted of mixing PMMA polymer powder with the liquid monomer. For each test, one ampoule of the liquid (20 mL) was added to one packet of the powder (40 g) in a plastic beaker. A spatula was used to stir the mixture per the manufacturer’s guidelines. The liquid mixture was drawn up into a 10 mL standard Luer-Loc syringe. The syringes were then maintained at the desired temperature. The cement was assessed to determine whether it could still be injected using a cement compressor [16]. By twisting the compressor, the piston delivers its axial load to the plunger, pumping out the bone cement (Figure 1). Cement injectability was tested by twisting the compressor two turns to pump the bone cement out. The amount of bone cement delivered in two consecutive twists was 0.3 mL. The bone cement was considered injectable when it could be pumped out through the syringe orifice in a liquid or paste form. The liquid time is defined as the period that the bone cement would drip from the orifice at the open end of the syringe when a slight torque is loaded on the compressor. The paste time is defined as the period that the bone cement can be ejected from the syringe without breaking its continuity (Figure 2). The handling time is the time that elapses after mixing the components until the bone cement cannot be pumped out from the syringe; the handling time is equal to the liquid time plus the paste time.

In the group 1 study (room temperature), the PMMA powder and liquid, as well as the beaker, spatula, and syringe were kept at ambient room temperature (22°C) before mixing the bone cement. The mixture was stirred for one min before testing. Cement injectability was tested every 30 s at room temperature. In group 2 (precooling the bone cement and the container), the PMMA powder and liquid, as well as the beaker, spatula, and syringe, were stored overnight in the refrigerator (4°C). The experiments were performed immediately upon removing the bone cement and the container from the refrigerator. The mixture was stirred for 2 min before testing. Injectability was tested every 30 s at room temperature. In group 3 (ice bath cooling), the PMMA powder and liquid, as well as the beaker, spatula, and syringe were kept at ambient room temperature (22°C) before mixing the bone cement. The mixture was stirred for one min before testing. The syringes were immediately submerged in a sterile ice water bath after mixing the bone cement at room temperature. A temperature of 0°C was achieved by mixing ice and water and was confirmed by a thermometer. Syringes were removed at 5-min intervals to assess injectability.

Eight repetitions of each group were performed, resulting in 24 trials. Photographs were taken to graphically demonstrate the viscosity at each injection. The parameters collected for analysis were the liquid, paste, and handling times. To evaluate the effects of the different cooling methods on the liquid time and paste time in specific situations, the time differences among the three groups were compared using an ANOVA test. The results were considered significant when p-values were less than 0.05.

## Results

**Figure 3:**
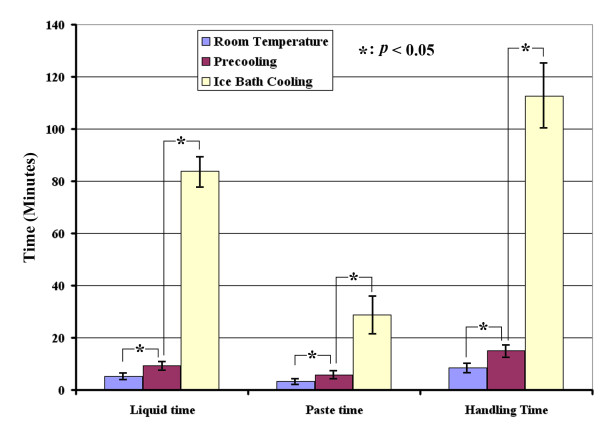
**The liquid time, paste time, and handling time of group 1 (room temperature, 22°C), group 2 (precooling), and group 3 (ice bath).** The liquid time and paste time of the two cooling methods are significantly (* *p*<0.05) longer than those of the control group (room temperature, 22°C). The liquid time and paste time of the ice bath method are significantly longer than those of the precooling method (* *p*<0.05).

The data for the liquid time, paste time, and handling time of the 3 groups are presented in Figure 3. Data are presented as the mean ± the standard deviation. In group 1 (room temperature, 22°C), the average liquid time was 5.1 ± 0.7 min, and the average paste time was 3.4 ± 0.3 min. The bone cement hardened at 8.5 ± 0.8 min. In group 2 (precooling the bone cement and the container), the average liquid time was 9.4 ± 1.1 min, and the average paste time was 5.8 ± 0.5 min. The bone cement hardened at 15.2 ± 1.2 min. In group 3 (ice bath cooling), the average liquid time was 83.8 ± 5.2 min, and the average paste time was 28.8 ± 6.9 min. The bone cement hardened at 112.5 ± 11.3 min.

The key finding of this study is that the liquid and paste times can be increased by different types of cooling methods (groups 2 and 3), when compared to mixing the liquid and the powder at room temperature (group 1). Additionally, the time intervals for ice bath cooling (group 2) are statistically longer than for the precooling study (group 3).

## Discussion

### Polymerization

Bone cements are usually supplied as two component systems, including a liquid and a powder. The powder primary consists of bead-shaped particles that are approximately 40 μm in diameter. These particles contain, in addition to methylmethacrylate copolymers, benzoyl peroxide BPO (the so-called initiator), and zirconium or barium to provide radio-opacity [17]. The second component of bone cement, the liquid, mainly contains the monomers. When the two components are mixed, polymerization is initiated, and self-curing occurs. At room temperature, monomer polymerization can be activated only in the presence of free radicals [18]. These radicals are produced during the reaction of the initiator, BPO, which is contained in the powder. Polymerization is an exothermic reaction, which means that it produces heat and is adversely affected by the application of heat [19,20]. Core temperatures of 77.3°C have been measured in the center of bone cement in an *in vitro* vertebroplasty study [20]. This temperature is above the coagulation temperature of proteins. Once polymerization ends, the temperature decreases and the cement becomes solid.

### The phases of bone cement

The handling of bone cement can be described by four different phases, based on the corresponding viscosities [18]. The first phase is the mixing phase (up to 1 min), which is the period required to thoroughly homogenize the powder and the liquid. The powder and the liquid can be mixed manually using a bowl and a spatula. Second, the waiting phase (up to several minutes, depending on the type of cement and the handling temperature) is the period required for the cement to reach a non-sticky state. Third, the working phase (2–4 min, depending on the type of cement and the handling temperature) is the period in which the cement is injected. Lastly, the hardening phase is a short period in which the final setting process occurs and polymerization heat develops. In this study, the liquid time is assumed to coincide with the duration of the waiting phase. The hardening phase is difficult to define in clinical situations; thus, the paste time is considered the duration of the working phase together with the hardening phase. Because the hardening phase is short, the paste time roughly corresponds to the working time.

An early injection in the liquid phase may result in bone cement extravasation into the venous system as well as its distant migration to the lungs [10,21]. If paravertebral vein filling is observed by fluoroscopy, the cement injection should be stopped and staged. Cement viscosity must also be sufficiently high to withstand the blood pressure. If blood mixes with the cement, its strength is reduced. However, a late injection of high viscosity bone cement may result in poor interfaces between the cement and the bone. Additionally, it is difficult to inject the cement through the cannula or spinal needles when it is approaching the final hardening stage.

### Arrhenius equation

Cement polymerization is an exothermic reaction [3]. The Arrhenius equation illustrates the exponential effect of temperature on the reaction [22]. In short, the Arrhenius equation gives “the dependence of the rate constant *k* of chemical reactions on the temperature T (in absolute temperature or kelvins) and activation energy Ea,” as shown below:

$$k=Ae^{-E_{a}/RT}$$

By lowering the temperature T, a decreased rate constant *k* can be expected. Thus, the polymerization time can be increased by lowering the temperature. The instructive package inserts in the commercially available products provide graphic information on the duration of each period with relation to temperature, which indicate that the working time is approximately 2–4 min, depending on the type of cement and the handling temperature. In this study, precooling (i.e., lowering the initial temperature) and ice bath cooling (i.e., lowering the surrounding temperature) prolonged the handling time to 15.2 min and to 112.5 min, respectively. Cooling the mixture is an important method of increasing the duration of injectability. As newer cements are developed, we believe that this general principle will remain the same.

### Precooling method

It is convenient to store the cement used for percutaneous vertebroplasty in a refrigerator before mixing to prolong the liquid and the paste times. In the current study, storing the PMMA (liquid ampoule and power packet) and the mixing and injection devices (plastic beaker, spatula, and syringe) in the refrigerator was found to effectively increase the liquid time and the paste time. Our experiment yielded a 1.9-fold increase in the liquid time, a 1.7-fold increase in the paste time and a 1.8-fold increase in the handling time, compared with the polymerization at room temperature. Refrigeration provides a convenient and accessible cooling method if ice is not readily available. Initially, the cement will be overly runny, and the clinician must assess its viscosity before further delivering it. By delaying delivery for a short time, the viscosity will increase until the cement reaches an adequate consistency. With refrigeration, more time will be available to monitor the process of cement distribution within the vertebrae. The procedure can be performed in a controlled manner without any added pressure due to time, and theoretically the possibility of cement leakage will be reduced [12,15].

### Ice bath method

After mixing the bone cement and filling the syringe at room temperature, the device was stored in ice water. The syringes were removed for a short period at 5-min intervals to assess the bone cement injectability. The liquid time and paste time increased dramatically, and we observed a substantial retardation of the polymerization process [12]. It has been recognized that placing a cement mixture in an ice bath has a significant influence on the cement polymerization time [12]. Chavali et al. [12] qualitatively investigated the extension of the polymerization time of bone cement with ice bath cooling. They concluded that the injectability of a PMMA mixture could be improved by cooling it in an iced bath. However, Chavali’s study only gave a qualitative description, without any quantitative data and statistical analysis. In the present study, not only the liquid, paste, and handling times were compared among three groups, the mechanism of extending polymerization time was also illustrated by Arrhenius equations. Our results indicated that even though the handling time increased by precooling method was less than ice bath cooling, precooling method is easier for application. Surgeons can choose either method according to different clinical needs.

Our experiment yielded a 16.5-fold increase in the liquid time, an 8.4-fold increase in the paste time and a 13.2-fold increase in handling time compared with the polymerization at room temperature. Long liquid times (or waiting times) allow the cement to be injected at a fairly constant consistency with one preparation, even when multiple spinal levels need vertebroplasty. In our previous study, the average amount of bone cement needed per vertebra was 4-6 mL [23,24]. The amount of bone cement made in each preparation could fill up two 10 ml syringes. By simultaneously submerging the two syringes in ice water, a clinician could successfully inject up to four vertebrae.

### Simple and decreases costs

The question of whether temperature alterations change the biomechanical properties of bone cement remains controversial [25-27]. Lewis [26] assessed the influence of the storage temperature of the unmixed cement constituents (21°C vs. 4°C) on the fatigue performance. They concluded that the storage temperature does not exert a significant influence on the fatigue performance of the bone cement. However, Vallo’s study [27] demostrated that decreasing the external temperature of bone cement will decrease the peak curing temperature, which will increase the amount of residual monomer present in the cement. This remaining unreacted monomer acts as a plasticizer, softening the cement. Additionally, some of the current clinical and biomechanical data suggest that vertebroplasty can cause the development of adjacent vertebral fractures shortly after augmentation [28,29]. These findings have been attributed to high Young’s moduli of PMMA bone cements compared to that of the osteoporotic cancellous bone. Although cooling the exterior of the cement might reduce its mechanical properties, this concern should not influence the method’s application in vertebroplasty because the gap between the mechanical strength of the bone cement and that of the osteoporotic cancellous bone is very large.

Some clinicians have routinely used temperature reduction methods in percutaneous vertebroplasty and have found no adverse side effects [8,15]. The increased handling time allows the clinician to leave the cement, which has filled the leak side or the paravertebral vein, to act as a plug before continuing the injection. The increased handling time provides the clinician with time to discern how the bone cement is filling the vertebral body. Cooling, especially the ice bath technique, is also a good method for training allowing multiple injections into different vertebrae from one preparation.

### Limitations

This report has some limitations. We only studied the effects of temperature reduction on one type of cement. There are many types of bone cement with different chemical and physical properties. Additionally, some clinicians prefer to use 1 mL syringes or plungers for cement injection instead of the 10 mL standard Luer-Loc syringes. The injectability of bone cement varies among different brands and devices. Surgeons who want to apply these hypothermic techniques have to set up their own protocols.

## Conclusions

The results of this study show that the polymerization or the curing interval of bone cement can be manipulated by altering the temperature. Both precooling (i.e., lowering the initial temperature) and ice bath cooling (i.e., lowering the surrounding temperature) methods can effectively increase the liquid time and the paste time. Precooling is an easy method for clinical applications, while ice bath cooling might be more suitable for multiple-level vertebroplasty. Through these methods, clinicians can take advantage of the increased handling time and the improved injectability without any increased cost.

## Misc

Po-Liang Lai and Ching-Lung Tai These authors contributed equally to this work

## Competing interests

This study was supported by a financial grant from Chang Gung Memorial Hospital (CMRPG381301) and the National Science Council (100-2622-E-182-004-cm^3^). The funding sources did not have any influence on the investigation.

## Authors’ contributions

PLL carried out the study and drafted the manuscript. CLT participated in the design of the study, the interpretation of the results and the draft of the manuscript. IMC initiated the concept of precooling the bone cement and set up the protocol. TSF participated in the design of the study and helped with the data collection. LHC participated in the design of the study and helped with the analysis of the data. WJC participated in carrying out the study and reviewing the references. All authors read and approved the final manuscript.

## Pre-publication history

The pre-publication history for this paper can be accessed here:

http://www.biomedcentral.com/1471-2474/13/198/prepub
